# Effectiveness of Omega-3 Fatty Acid Supplementation in Improving the Metabolic and Inflammatory Profiles of Mexican Adults Hospitalized with COVID-19

**DOI:** 10.3390/diseases12010028

**Published:** 2024-01-17

**Authors:** Diana Rodríguez-Vera, Juan Rodrigo Salazar, Marvin A. Soriano-Ursúa, Jessica Guzmán-Pérez, Arely Vergara-Castañeda, Horacio Muñoz-Durán, Gabriela L. Ramírez-Velez, Alonso Vivar-Sierra, Carlos Rogelio Naranjo-Navarro, Patricia A. Meza-Meneses, Marco A. Loza-Mejía, Rodolfo Pinto-Almazán

**Affiliations:** 1 Sección de Estudios de Posgrado e Investigación, Escuela Superior de Medicina del Instituto Politécnico Nacional, Plan de San Luis y Diaz Mirón s/n, Mexico City 11340, Mexico; drodriguezv1904@alumno.ipn.mx (D.R.-V.); msoriano@ipn.mx (M.A.S.-U.); jess.gupe@gmail.com (J.G.-P.); 2 Design, Isolation, and Synthesis of Bioactive Molecules Research Group, Chemical Sciences School, Universidad La Salle-México, Benjamín Franklin 45, Mexico City 06140, Mexico; juan.salazar@lasalle.mx (J.R.S.); gabriela.ramirez@lasalle.mx (G.L.R.-V.); alonso.vivar@lasallistas.org.mx (A.V.-S.); cr.naranjo@lasallistas.org.mx (C.R.N.-N.); 3 Promotion and Education for Health and Food Research Group, Chemical Sciences School, Universidad La Salle-México, Benjamín Franklin 45, Mexico City 06140, Mexico; arely.vergara@lasalle.mx; 4 Environmental Technology Division and Section of Postgraduate Studies, Universidad Tecnológica de Nezahualcóyotl. Cto. Rey Nezahualcóyotl Manzana 10, Benito Juárez, Nezahualcoyotl 57000, Mexico; horaciomunozdu@utn.edu.mx; 5 Servicio de Infectología, Hospital Regional de Alta Especialidad de Ixtapaluca, Ixtapaluca 56530, Mexico; patricia_meza@ymail.com

**Keywords:** COVID-19, omega-3, polyunsaturated fatty acids, nutrition, diet

## Abstract

Background and Objectives: The development of severe COVID-19 is related to the preexistence of comorbidities and an inadequate nutritional status. The latter is a critical factor for the development of infection and the progression of the disease. Notably, optimal nutrition impacts immune system function, as malnutrition is related to high cytokine levels in the late phase of the disease, correlating with a poor prognosis. In this sense, omega-3 fatty acids (O3FAs) have anti-inflammatory properties that may reduce morbidity and mortality from COVID-19 infection. O3FAs are linked to a better prognosis in COVID-19 patients. Materials and Methods: In this randomized, double-blind clinical trial, we evaluate the administration of O3FAs to unvaccinated Mexican patients for two weeks starting after the first two hours of hospitalization. Results: The findings support the notion that O3FAs (in a dose high enough to satisfy human physiological requirements in a short time, one capsule of 1.4 g O3FAs daily) exert a comprehensive multi-systemic modulatory influence, affecting inflammatory and metabolic pathways. Significant perturbations in biomarkers, including absolute neutrophil count, hematocrit, and platelet indices, underscore the compound’s anti-inflammatory effect. Concurrently, the intervention modulates pivotal metabolic and hepatic parameters, attenuating cardiovascular risk profiles and expediting patient convalescence. These multifarious effects are likely orchestrated through intricate biochemical mechanisms and are subject to individual variations predicated on metabolic factors. Conclusions: The results of this trial support the notion that O3FA supplementation has beneficial effects on COVID-19 patients with moderate presentation by regulating metabolism and limiting inflammation.

## 1. Introduction

Coronavirus disease 2019 (COVID-19) is the result of infection by severe acute respiratory syndrome coronavirus 2 (SARS-CoV-2) [1]. This disease caused almost seven million deaths in three years, posing a global health emergency in 2020 [2,3,4,5,6]. COVID-19 symptoms in patients range from nonexistent to severe. Severe complications can overdrive the host immune system, leading to a “cytokine storm” [7]. Severe infections are related to comorbidities, such as obesity, diabetes, chronic obstructive pulmonary disease, chronic heart failure, coronary artery diseases, metabolic syndrome, and hypertension [8]. In this regard, Galindo-Oseguera et al. identified an increased risk of mortality in the presence of hypertension and diabetes linked to an exacerbated inflammatory profile in moderate and severe pneumonia due to COVID-19 in patients attending the High Specialty Regional Hospital of Ixtapaluca, Mexico (HRAEI), a regional hospital [9]. SARS-CoV-2 infection causes a pattern of metabolic and clinical manifestations, leading to leukocytopenia, lymphopenia, and elevated levels of C-reactive protein (CPR) in patients with the primary form of COVID-19 [1,10,11,12].

In addition, malnutrition is linked to an inadequate immune response to viral infections [10,13,14]. Specifically, in COVID-19, nutritional deficiencies may lead to increased oxidative stress in the host [10,15]. Therefore, nutritional status is a critical factor in moderating the susceptibility to and progression of COVID-19 [2,16,17]. In this regard, omega-3 polyunsaturated fatty acids (O3FAs) are essential components of a heathy diet. In fact, O3FAs have the potential to reduce COVID-19 susceptibility and severity [2,18,19,20,21,22]. The metabolites of O3FAs play an essential role in the synthesis of different inflammatory mediators from the cellular membrane [23], as well as improving macrophage function [24]. Thus, O3FAs have immunomodulatory effects on viral infection and tissue damage [2,25,26,27]. In this way, severe cases of COVID-19 are related to hyperinflammation [28], while O3FA administration is related to lower risks of requiring mechanical ventilation and death [29]. Moreover, O3FAs are known for their ability to downregulate the inflammatory response induced by the innate immune system [2]. In this sense, Hathaway et al. studied how O3FAs from fish oil enhanced the antiviral response by inducing interferon production, leading to the inhibition of viral replication, while ameliorating the response of CD8 T cells involved in unintended lung damage and further deteriorating the clinical outcome [30,31].

Thus, it was proposed that increasing O3FA consumption reduces viral entry, ameliorates immune function, and diminishes the severity of some COVID-19 complications. Sun et al. observed that O3FAs are inversely associated with the risk of severe COVID-19. Specifically, they found that O3FAs and docosahexaenoic acid (DHA) measured in either plasma or red blood cells were inversely associated with COVID-19 susceptibility and severity [2]. The administration of these fatty acids is considered a safe and inexpensive prophylactic treatment approach for high-risk people [32,33,34]. Although the mechanisms remain to be elucidated, it is known that O3FAs are widely distributed in the body for further oxidation, storage, or metabolism [34]. However, they are taken up by neutrophils to produce inflammation mediators [35,36]. Indeed, O3FAs reduce neutrophil infiltration, pro-inflammatory mediators, and classical monocytes, and enhance non-classical monocyte/macrophage recruitment in sepsis [37,38].

Scarce data have been reported about the specific nutrients associated with protective action in the Mexican population with COVID-19, even if some studies suggest the supply of food as a protective strategy [39]. Therefore, the present study aimed to evaluate for first time the effects of O3FA supplementation in unvaccinated Mexican patients with COVID-19, focusing on biochemical/clinical parameters and inflammation markers in moderate and severe COVID-19 patients treated at the HRAEI.

## 2. Materials and Methods

### 2.1. Study Design and Population

This study was a double-blind, randomized clinical trial performed from May to July 2021 in moderately ill patients infected with COVID-19 at the HRAEI, Mexico. Capsules filled with corn oil were used as a placebo (very similar in appearance to those used in the intervention group with O3FAs), but patients were unaware of their feeding contents. However, patients and researchers needed to be made aware of the arms of the study. The results were analyzed by an independent evaluator who was not a member of the treatment team. The sample size was calculated using a proportion difference formula with 95% accuracy and 80% power, as well as the results of a previous report by Kosmopoulos, in which an early anti-inflammatory effect among symptomatic outpatients with COVID-19 was observed in 52% of patients supplemented with O3FAs vs. 24% of control patients, resulting in the determination of the number of patients per group. Recruited patients were randomized into either the group receiving O3FA supplementation or the control group through the generation of random numbers, as described in [40].

Patients were recruited from the emergency room of the hospital considering the following inclusion criteria: aged 18 or older and diagnosed with COVID-19 via a positive RT-PCR nasopharyngeal swab. Clinical evaluation and the CO-RADS classification was used to determine moderate disease (Supplementary Table S1) [41]. Briefly, participants were included if they exhibited clinical manifestations such as severe and intermediate pneumonia, fever, fatigue, dry cough, and respiratory distress requiring hospitalization. Requiring oxygen therapy was also a requisite for inclusion. Moreover, participants were included if they had at least one underlying comorbidity, such as diabetes, obesity, or hypertension. Eligibility was further restricted to those indicated for enteral nutrition.

The exclusion criteria included: (a) patients who were either intubated or at a significant risk of imminent intubation; (b) patients with a history of severe hemorrhagic disorders and those with previous reports of myocardial infarction, acute shock, or comatose states; (c) patients who consumed O3FAs during the three months prior to the study; (d) patients with a history of hypersensitivity reactions to fish or its products; and (e) patients who started a vaccination scheme (as the immune response could induce confusing changes). Participants who could not complete the study due to mortality or the cessation of the need for enteral feeding were also excluded.

In the present study, 40 patients infected with any variant of COVID-19 were selected using the inclusion criteria mentioned above, of whom 23 made up sample one (n1) and were not supplied with O3FAs. However, sample two (n2) comprised 17 patients who ingested O3FAs orally for two weeks. Although the sample size was not equal between groups, a per-protocol analysis was performed to evaluate the impact of supplementation up to that point, resulting in the identification of significant clinical differences. 

The intervention group received one capsule of 1.4 g O3FAs daily (GNC triple-strength fish oil), containing 316 mg of eicosapentaenoic acid (EPA) and 381 mg of DHA, added to their initial treatment. This dose was considered in accordance with the dietary recommendations of over 50 organizations described in the Global Recommendations for EPA and DHA Intake (500 mg/person/day) and the U.S. Department of Health and Human Services and the U.S. Department of Agriculture 2015–2020 [42,43]. O3FAs were administered to the case group (n2), and the placebo was administered to the control group (n1) by a physician for two weeks, starting after the first two hours of hospitalization.

### 2.2. Data Collection 

After collecting written consent forms, data were systematically gathered from electronic medical records. Demographic and clinical variables were meticulously extracted from these records, including age, prior medical conditions, medical history, blood pressure metrics, serum lipid profiles, random blood glucose levels, and respiratory status. 

In order to rigorously assess both biochemical and pathological markers, an exhaustive battery of diagnostic assays was performed, which included the evaluation of arterial blood gas metrics, such as oxygen saturation (O_2_ Sat) and arterial pH, as well as renal function indices encompassing blood urea nitrogen (BUN), creatinine (Cr), and urinary output volume. Glycemic profiles, mean arterial pressure, and a complete hematological panel—comprising differential leukocyte counts (neutrophils, lymphocytes, and monocytes)—were also scrutinized. Supplementary parameters, including the Glasgow Coma Scale, hemoglobin concentration, platelet count, and activated partial thromboplastin time, were likewise subjected to rigorous analysis. Serum albumin concentrations and hematocrit levels were quantified as ancillary markers. These multifaceted evaluations were conducted at baseline and after the 14-day interventional period. All assays were performed in strict compliance with institutional protocols, utilizing standardized reagent kits, and the institution’s laboratory division meticulously collated the resultant data.

### 2.3. Statistical Analysis

Descriptive statistics were employed to determine the nominal characteristics of the patients. To identify whether the differences between “before” and “after” for each variable had a normal distribution, the Shapiro–Wilk normality test for continuous variables was used, with a significance level of 5%. Since the data came from paired or related samples, this procedure was conducted prior to the analysis of the information collected from the laboratory. Then, a hypothesis was formulated according to the test statistics; the differences in sample means were compared with the t-Student statistic (with normality in the differences), and the non-parametric Wilcoxon test (without normality in the differences) was used to determine whether the differences between the medians were equal to or different from zero. *p*-values less than 0.05 were considered statistically significant. Statistical analyses were performed using IBM SPSS software (version 25, 2017, Armonk, NY, USA: IBM Corp.).

## 3. Results

### 3.1. Demographic Results 

The average age of the patients in group n1 was 52.1 years, ranging from 26 to 81, and the average hospital stay was 10 days, ranging from 2 to 38 days. Meanwhile, the average age of the patients in group n2 was 48.8 years, ranging from 25 to 82. The mean duration of hospitalization was 5 days, ranging from a minimum of 2 days to a maximum of 8 days for patients requiring the most extensive medical care. All patients were from State of Mexico, Mexico City, and the metropolitan region (including states in the center of the country). No demographic differences were notable between the two groups, neither in the comorbidity nor in the severity of included patients (Supplementary Table S1).

### 3.2. Effects of O3FA Supplementation on Measured Markers

**Figure 1:**
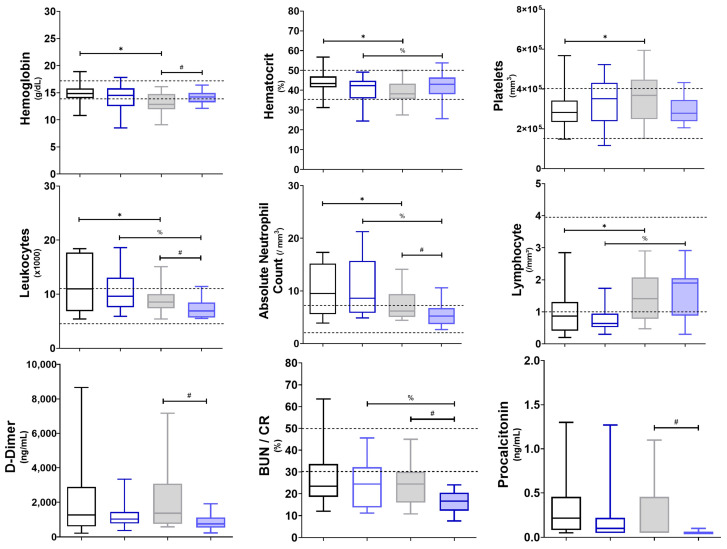
Blood biometry cell counts pre- and post-treatment. Markers of infection, inflammation, and tissue damage. Gray boxes represent untreated patients (n1), while blue boxes represent patients supplemented with O3FAs (n2); the left (blank) shows individuals pre-treatment, and the right (filled) shows individuals post-treatment. *^,#,%^ *p* < 0.05 against the group indicated in the extremes of the horizontal bars: * *p* < 0.05 between before and after the study period in the untreated group; ^%^ *p* < 0.05 between before and after the study period in the supplemented group; ^#^ *p* < 0.05 between the final state of both the treated and untreated groups. Abbreviation: BUN/CR = Blood Ureic Nitrogen/Creatinine ratio.

O3FA supplementation led to a significant reduction in leukocyte counts, particularly neutrophils, and a decrease in hematocrit levels, as depicted in Figure 1. The homogenization of the inflammatory response post-O3FA supplementation, as evidenced by the reduced variability in the absolute neutrophil count and hematocrit levels (Figure 1), suggests a modulatory effect on neutrophil function. 

**Figure 2:**
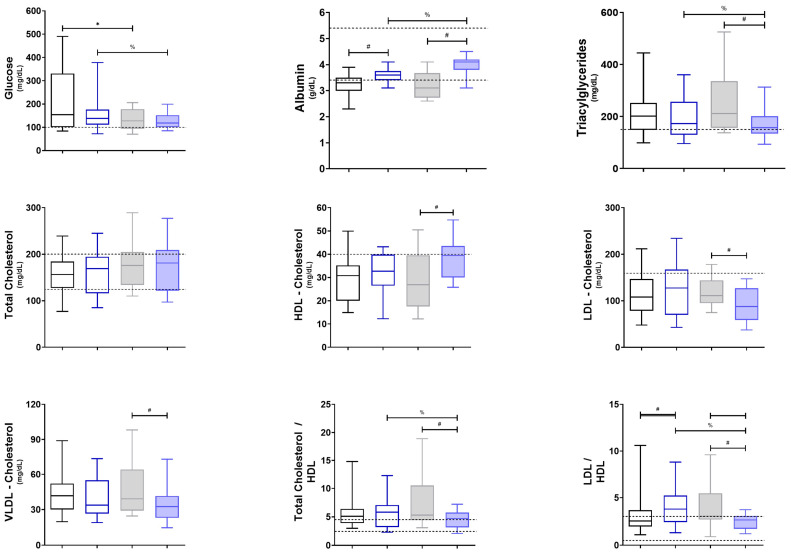
Markers of general metabolism. Gray boxes represent untreated patients (n1), while blue boxes represent patients supplemented with O3FAs (n2); the left (blank) shows individuals pre-treatment, and the right (filled) shows individuals post-treatment. *^,#,%^ *p* < 0.05 against the group indicated in the extremes of the horizontal bars. HDL = High-Density Lipoproteins, LDL = Low-Density Lipoproteins, VLDL = Very Low-Density Lipoproteins.

Regarding metabolism markers (Figure 2), the post-treatment lipid profile exhibited nuanced alterations with implications for cardiovascular health. A significant increase in high-density lipoprotein (HDL) cholesterol, with a correlation with the decrease in total cholesterol/HDL and LDL/HDL ratios, was observed after O3FA treatment compared to the control group (Figure 2). In contrast, low-density lipoprotein (LDL) and very-low-density lipoprotein (VLDL) cholesterol levels showed significant reductions, with the former displaying a positively skewed distribution, indicating a potentially more significant impact on individuals with elevated baseline VLDL and LDL levels. Triglyceride levels also demonstrated a noteworthy decline, a finding of substantial clinical relevance given the role of triglycerides as a cardiovascular risk marker.

Concomitantly, there were marked reductions in glucose, creatinine, and BUN levels, indicative of a broader metabolic modulation. Most notably, the observed decreases in the LDL/HDL and total cholesterol/HDL ratios carry clinical significance. Elevated ratios are conventionally viewed as risk factors for cardiovascular diseases associated with severe COVID-19 outcomes. 

**Figure 3:**
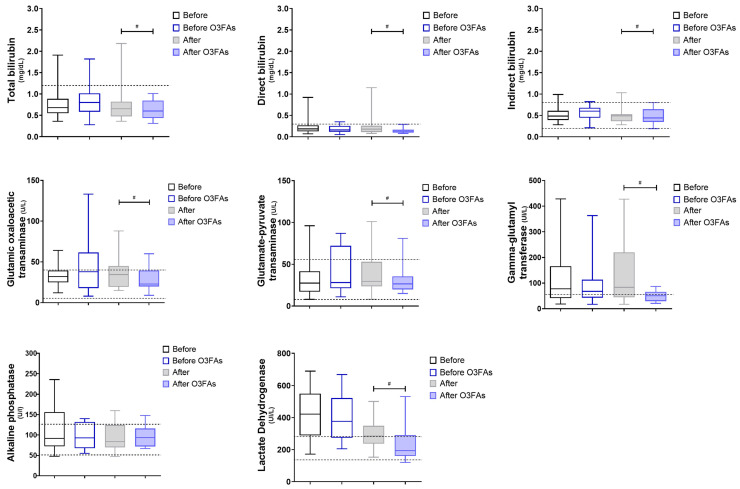
Liver function. Metabolites and enzymes as markers of liver function. Gray boxes represent untreated patients (n1), while blue boxes represent patients supplemented with O3FAs (n2); the left (blank) shows individuals pre-treatment, and the right (filled) shows individuals post-treatment. ^#^ *p* < 0.05 against the group indicated in the extremes of the horizontal bars.

Glutamate-pyruvate transaminase (GPT) showed increased variability coupled with a narrowed interquartile range, alluding to a complex influence on this enzyme (Figure 3). Concurrently, glutamic oxaloacetic transaminase (GOT) exhibited a decline characterized by a positively skewed distribution, suggesting a potential asymmetric therapeutic benefit. Alkaline phosphatase showed greater data dispersion, necessitating further exploration of its mechanistic underpinnings. 

Finally, both direct and indirect bilirubin levels demonstrated reduced variability, underscoring stabilization in hepatic excretory function. Additionally, it should be mentioned that lactate dehydrogenase (LDH) has been used as a marker of cellular damage and is elevated in severe COVID-19 cases. The administration of O3FAs, specifically EPA and DHA, has been observed to attenuate the levels of these biomarkers significantly. This reduction suggests a systemic modulation of both the inflammatory and coagulative responses, potentially mediated through the inhibition of pro-inflammatory eicosanoids and alterations in inflammation-related gene expression profiles. The observed asymmetric distribution in the reduction in LDH levels post-O3FA administration adds a layer of complexity, implying a non-linear dose–response relationship and suggesting that therapeutic efficacy may be more pronounced in specific subpopulations, such as those with initially elevated LDH levels or those at a more advanced stage of the disease.

## 4. Discussion

Our study elucidates the multifaceted impact of O3FA supplementation on systemic inflammation, coagulation, and metabolic modulation in unvaccinated COVID-19 patients [44]. The identification of O3FA-administration effects on factors that increase or limit the risk of developing severe forms of COVID-19 is particularly noteworthy [45].

The observation findings in the present study are broadly consistent with those in previous observational studies, in which O3FAs were found to be inversely associated with the risk of severe COVID-19 when comparing patients to individuals with an unknown COVID-19 status [45,46,47]. However, this is the first time in an unvaccinated Mexican population, which showed highly incidence of severe cases and mortality.

This study delineates the multifarious ramifications of O3FA supplementation on the immunological and metabolic landscape in COVID-19 patients with a population-recommended dose aimed to satisfy human physiological requirements in a short time [45,46,47,48,49,50]. The data substantiate the nuanced modulation of key inflammatory and coagulative markers. In fact, data in Figure 1 can be interpreted as a systemic attenuation of the inflammatory response, a critical factor given the role of cytokine storms and acute inflammation in COVID-19 morbidity and mortality. Concurrently, the metabolic perturbations induced by O3FA supplementation result in lipidomic realignments and enzymatic variabilities, with notable implications for health. These findings corroborate the extant literature and augment our understanding of the mechanistic underpinnings of O3FA-mediated modulations in the context of a viral pandemic [45,50,51,52].

Regarding inflammation and complication factors, the administration of O3FAs led to a notable decrease in neutrophil counts, possibly indicative of a systemic downregulation of the NLRP3 inflammasome. This effect is likely mediated through biochemical pathways that inhibit pro-inflammatory eicosanoid synthesis, alter gene expression related to inflammatory mediators, and modify cell membrane characteristics affecting signal transduction. It could mitigate the cytokine storm frequently observed in severe COVID-19 cases, related to disseminated intravascular coagulation, acute respiratory distress syndrome, multiple organ dysfunction syndrome, and death [46]. Hemoglobin levels also exhibited a significant decrease post-O3FA supplementation, which could be attributed to the anti-inflammatory properties of O3FAs affecting erythropoiesis [47,51]. Additionally, well-established biomarkers, such as procalcitonin, D-dimer, and the BUN/CR ratio, were attenuated post-O3FA administration, signifying a systemic modulation of both inflammatory and coagulative responses [48,49,50,51]. It should be seen that D-dimer and procalcitonin serve as well-established biomarkers for infections, systemic inflammation, and coagulation events, and their elevated levels, as well as an elevated BUN/CR ratio, have been correlated with worse outcomes in COVID-19 patients [48,51].

The data also revealed intriguing distributional characteristics in lymphocyte and platelet counts, suggesting a systemic anti-inflammatory effect and potential antithrombotic properties [52]. The negative skewness in lymphocyte and platelet counts post-supplementation implies a concentration of data points on the higher and lower ends of the respective scales, signifying not only a systemic anti-inflammatory effect but also potential antithrombotic properties, given the role of platelets in coagulation and thrombosis. 

The high variability in platelet counts pre-O3FA supplementation limits us to suggesting a heterogeneous response. This is possibly attributable to individual metabolic, absorptive, or kinetics factors affecting fatty acid utilization and platelet formation.

From a clinical standpoint, these findings could translate into expedited patient recovery and reduced morbidity, potentially mitigating complications, such as thromboembolic events and acute respiratory distress syndrome (ARDS).

The role of O3FAs in modulating inflammation, cellular membrane fluidity, and macrophage function is particularly rich and nuanced [45,48]. They can also reduce the risk of SARS-CoV-2 infection by suppressing and inhibiting the progression of viral infections [45] and the development of critical symptoms [47]. 

The administration of O3FAs, specifically EPA and DHA, has elucidated attractive immunomodulatory effects, including those that enhance the response to viral infections [19,36,45,46]. The observed leukopenia, particularly the significant decrease in neutrophil counts, may indicate a systemic downregulation of the NLRP3 inflammasome, a cytosolic complex pivotal in initiating the inflammatory cascade, attenuating the release of pro-inflammatory cytokines, such as IL-1β and IL-18, thereby mitigating the cytokine storm frequently observed in severe COVID-19 cases [29]. The role of O3FAs in modulating Toll-like receptor (TLR) signaling pathways, which are integral to innate immunity, also warrants further investigation [45]. Furthermore, eicosanoids derived from EPA as resolvins, maresins, lipoxins, and protectins, inhibit leukocyte infiltration to the site of inflammation and stimulate macrophages and neutrophils to resolve inflammation, thereby averting prolonged symptomatology [45].

The post-supplementation homogenization of inflammatory markers, such as the absolute neutrophil count and hematocrit levels, underscores a nuanced modulatory effect on neutrophil function [52].

Even hemoglobin, a quintessential biomarker for oxygen transport, exhibited a notable decrease post-O3FA supplementation. This finding could be interpreted through multiple lenses. Firstly, O3FAs are known for their anti-inflammatory properties, and inflammation modulates erythropoiesis, producing new erythrocytes. Reducing inflammation could decrease the levels of erythropoietin, a hormone that stimulates erythropoiesis, thereby affecting hemoglobin concentrations. Secondly, O3FAs have been shown to modulate lipid membranes and could affect the integrity and function of red blood cell membranes, influencing hemoglobin levels [45].

Metabolically, significant alterations were observed in lipid profiles and other metabolic markers. O3FAs have been reported to enhance endothelial function and microcirculation, thereby optimizing blood flow and tissue perfusion. Significant elevations were observed in HDL cholesterol, while reductions were noted in LDL and VLDL cholesterol levels. Marked reductions were also observed in metabolic markers, such as glucose, creatinine, and BUN, indicative of broader metabolic modulation [53].

Thus, lipid profiles exhibited nuanced alterations with implications for cardiovascular health, including a significant elevation in HDL cholesterol and reductions in LDL and VLDL cholesterol levels. The observed decreases in the LDL/HDL and total cholesterol/HDL ratios not only suggest a cardio-protective effect but also contribute to an overall improvement in the patient’s clinical status [43,44].

O3FAs have been reported to enhance endothelial function and microcirculation, optimizing blood flow and tissue perfusion to prevent the progression of hypoxia and organ dysfunction. They modulate vasodilatory and vasoconstrictive responses, thereby enhancing microvascular function [32,48].

The attenuation of well-established biomarkers, such as procalcitonin, D-dimer, and LDH, post-O3FA administration signifies a systemic modulation of inflammatory and coagulative responses. The asymmetric distribution in reducing LDH levels adds a layer of complexity, suggesting a non-linear dose–response relationship and indicating that therapeutic efficacy may be more pronounced in specific subpopulations [32,48].

Concurrently, there were marked reductions in metabolic markers, such as glucose, creatinine, and BUN, indicative of broader metabolic modulation. Enzymatic markers such as GPT and GOT exhibited complex variability and distributional characteristics, necessitating further mechanistic exploration. Additionally, the significant elevation in albumin levels and total protein may imply improvements in nutritional status or hepatic function, corroborated by stabilized bilirubin levels [45]. Adequate albumin levels appear to be key since albumin displays antioxidant properties, such as scavenging oxygen free radicals, and the COVID-19 patients with higher albumin levels on admission were associated with a better overall prognosis [54]. However, further approaches should be carried out to confirm or discard these possibilities. 

In summary, the administration of O3FAs demonstrated profound modulatory effects on both immunological and metabolic parameters. These multifaceted changes could collectively influence systemic inflammatory responses, cellular signaling mechanisms, and overall organ functionality [45]. Further studies are needed to confirm these findings and explore the dose–response relationship [50].

Moreover, the effects of omega-3 supplementation after 14 days were observed in inflammatory parameters related to immunity. This trial supports the hypothesis that O3FA supplementation with a modest dose of at least near 1 g/day of EPA + DHA has significant benefits, consistent with those reported in other pathologies, and it also positively affected the survival rate of ill patients with COVID-19 [49]. This could be explained, as some studies have shown that participants with lower intake or baseline levels of O3FA, such as the Mexican population, are prone to having more impactful results after supplementation [50]. 

Given the high public health concerns related to the COVID-19 pandemic, modifiable risk factors for developing severe and critical complications, particularly nutritionally based O3FAs, were considered for their potential mechanisms underlying multiple actions [11].

The present study had some limitations. Samples were taken from patients in the center of the country, while diversity in the measured variables could be higher when using a multicentric approach. Despite the sample size being calculated with an acceptable power for the study, the results must be confirmed in more extensive studies due to small samples could increase the risk of fails in randomization processes. Although the differences found in this study suggest a superior effect of the intervention, only one O3FA dosage was studied, so the dose–response efficacy of the supplement was not analyzed, and the monitoring of some active compounds from diet intake as confounding factors was not carried out. Another limitation of this study is its short duration and the lack of standardized medical treatment. Furthermore, somatometry and biochemical markers linked to prognosis in COVID-19, such as body mass index, inflammatory cytokines (C-reactive protein (CRP)), and specific cytokines (interleukin-6 and interleukin-10, suggested as relevant biomarkers in predictive models of hospitalized COVID-19 patients [43,44]), were not measured mainly due to limited resources. Also, additional approaches are required to evaluate the effects of O3FA in vaccinated patients, including specific responses to diverse vaccines and vaccination schemes.

When comparing our findings with those of previous studies, it was found that the role of O3FAs in modulating inflammation and metabolic parameters (often measured and reported in an independent manner) was consistent with that observed in earlier research. Previous studies have shown that O3FAs were inversely associated with the risk of COVID-19 when comparing patients to individuals without COVID-19 [13]. Our study supports the hypothesis that O3FA supplementation positively affects the survival rate of ill patients with COVID-19 [50].

Given the high public health concerns related to the COVID-19 pandemic, identifying modifiable risk factors for developing severe and critical complications is crucial. In this context, O3FAs offer a nutritionally based option for their potential mechanisms underlying multiple actions. In this sense, previous studies in Mexico have indicated the leading risk factors for mortality in middle-aged COVID-19 patients: male, hypertension, drug addiction, and alcoholism [9,51,52]. However, scarce data support the beneficial effects of specific drugs or supplements in Mexican COVID-19 patients, triggering misconduct in drug availability, marketing, and application [53].

## 5. Conclusions

The administration of O3FAs, specifically EPA and DHA, in the context of COVID-19 has illuminated new avenues for therapeutic intervention, transcending the traditional boundaries of nutritional supplementation.

Our results support the well-known immunosuppressive effects of O3FAs, while the nuanced alterations in leukocyte counts are solely in short supply. The downregulation of neutrophils and the concomitant elevation of lymphocytes may signify a shift from innate to adaptive immunity. Furthermore, the observed improvements in lipid profiles and liver function tests suggest that O3FAs may exert a synergistic effect, modulating inflammatory responses and key metabolic indexes, raising the possibility of using O3FAs as part of a multi-pronged therapeutic strategy, targeting not only the virus but also the host’s metabolic and immune responses.

The data obtained in this study not only enrich our understanding of these fatty acids with multifaceted roles but also challenge us to reevaluate the existing paradigms in immunology and metabolic regulation by fatty acids. In addition, these data support the positive effects of O3FA supplementation in unvaccinated Mexican patients, as expected with multiple studies supporting biological effects related to potential benefits in COVID-19 treatment. The findings of this study have far-reaching clinical implications. Advanced analytical techniques, such as proteomics, metabolomics, and transcriptomics, could offer a more granular understanding of the molecular mechanisms. 

Likewise, further research employing rigorous methodologies, such as randomized controlled trials and multi-omics analyses, is imperative for elucidating the underlying biochemical and biomolecular mechanisms. This could pave the way for targeted therapeutic interventions and personalized medicine approaches in managing COVID-19, its complications, and other inflammatory diseases [46].

## Data Availability

Any additional data from this study can be requested by e-mailing the corresponding authors.

## Supplementary Materials

The following supporting information can be downloaded at: https://www.mdpi.com/article/10.3390/diseases12010028/s1, Table S1: Demographic, comorbidity, and severity (CO-RADS) of studied patients.
