# Supplementary material for: Effectiveness of Omega-3 Fatty Acid Supplementation in Improving the Metabolic and Inflammatory Profiles of Mexican Adults Hospitalized with COVID-19
Source: Diseases. 2024 Jan 17;12(1):28. doi: 10.3390/diseases12010028 (PMC10814050; doi:10.3390/diseases12010028)
Supplement: Supplementary file 1 [file diseases-12-00028-s001.zip › diseases-2809958-supplementary.pdf]

# Supplementary material

**Effectiveness of omega-3 fatty acid supplementation for improving the metabolic and inflammatory profiles of Mexican adults hospitalized with COVID-19. Diana Rodríguez-Vera** et al.

**Table S1.** Demographic, comorbidity and severity (CO-RADS) of studied patients.

**Control group (n1)**

| **Patient** | **Age** | **Gender** | **Overweight or obesity**[1] | **Diabetes** | **Hypertension** | **Dyslipidemia**[2] | **CORADS**[3] |
|---|---|---|---|---|---|---|---|
| 1 | 52 | F | YES | YES | YES | YES | 5 |
| 2 | 65 | F | YES | NO | NO | YES | 5 |
| 3 | 49 | M | YES | NO | NO | NO | 5 |
| 4 | 53 | M | YES | NO | NO | NO | 5 |
| 5 | 31 | M | YES | NO | NO | YES | 5 |
| 6 | 79 | M | YES | YES | NO | YES | 5 |
| 7 | 35 | F | YES | YES | NO | YES | 5 |
| 8 | 34 | M | YES | NO | YES | YES | 5 |
| 9 | 56 | M | YES | YES | NO | YES | 5 |
| 10 | 26 | M | YES | YES | NO | YES | 5 |
| 11 | 59 | M | YES | YES | NO | NO | 5 |
| 12 | 65 | M | YES | YES | NO | NO | 5 |
| 13 | 42 | M | YES | NO | YES | YES | 5 |
| 14 | 42 | M | YES | YES | NO | YES | 5 |
| 15 | 28 | M | YES | YES | YES | YES | 5 |
| 16 | 50 | M | YES | YES | NO | YES | 5 |
| 17 | 69 | F | YES | YES | YES | YES | 5 |
| 18 | 48 | M | YES | YES | NO | YES | 5 |
| 19 | 60 | F | YES | YES | NO | NO | 5 |
| 20 | 31 | M | YES | YES | NO | YES | 5 |
| 21 | 75 | F | YES | NO | NO | YES | 5 |
| 22 | 69 | F | YES | NO | NO | NO | 5 |
| 23 | 81 | F | YES | YES | NO | YES | 5 |
| Mean or percentage | 52.1 | M:65.2% F:34.8% | YES: 100% | YES:65.2% | YES:21.7% | YES:73.9% | 5 |

**O3FA supplemented group (n2)**

| Patient | Age | Gender | Overweight or obesity[1] | Diabetes | Hypertension | Dyslipidemia[2] | CORADS |
|---|---|---|---|---|---|---|---|
| 1 | 52 | M | YES | NO | NO | YES | 5 |
| 2 | 45 | M | YES | YES | NO | YES | 5 |
| 3 | 61 | F | YES | YES | YES | YES | 5 |
| 4 | 39 | F | YES | YES | NO | YES | 5 |
| 5 | 26 | F | YES | NO | NO | NO | 5 |
| 6 | 46 | M | YES | YES | NO | YES | 5 |
| 7 | 25 | M | YES | NO | NO | YES | 5 |
| 8 | 38 | F | YES | YES | YES | YES | 5 |
| 9 | 47 | F | YES | YES | NO | YES | 5 |
| 10 | 39 | M | YES | NO | NO | NO | 5 |
| 11 | 53 | F | YES | YES | NO | YES | 5 |
| 12 | 39 | M | YES | YES | NO | YES | 5 |
| 13 | 59 | M | YES | YES | NO | NO | 5 |
| 14 | 53 | M | YES | YES | NO | YES | 5 |
| 15 | 82 | M | YES | YES | NO | NO | 5 |
| 16 | 53 | F | YES | NO | YES | YES | 5 |
| 17 | 73 | M | YES | NO | NO | YES | 5 |
| Mean or Percentage | 48.8 | M:64.7% F:35.3% | YES:100% | YES:64.7% | YES:17.6% | YES:76.5% | 5 |

[1] Body Mass Index ≥25

[2] Triglycerides ≥150 mg/dL, and/or Low-Density Lipoproteins ≥190 mg/dL.

[3] CO-RADS was declared as a 5 value as imageology approach was very high suspicion of COVID-19. However, a PCR(+)-Test was obtained in all cases (corresponding to CO-RADS 6 in the original work [42]).
